# Chromosomal Abnormalities in Recurrent Pregnancy Loss at a Tertiary Care Center

**DOI:** 10.7759/cureus.93747

**Published:** 2025-10-02

**Authors:** Manisha B Sinha, Pushpawati Thakur, Renu Verma

**Affiliations:** 1 Anatomy, Lab of Cytogenetics and Molecular Reproduction, All India Institute of Medical Sciences, Raipur, Raipur, IND; 2 Obstetrics and Gynaecology, All India Institute of Medical Sciences, Raipur, Raipur, IND; 3 Nursing, All India Institute of Medical Sciences, Raipur, Raipur, IND

**Keywords:** chromosomal abnormalities, chromosome 9 heteromorphism, karyotyping, recurrent abortion, recurrent pregnancy loss, translocation

## Abstract

Background: Structural and numerical variation in chromosomes in couples leads to recurrent pregnancy loss/abortions, which disrupts the normal embryonic development. This study aimed to evaluate the incidence of chromosomal abnormalities in couples experiencing recurrent abortions.

Materials and methods: Thirty couples (N = 60) experiencing one or more abortions were enrolled. Couples experiencing one abortion were also included because of having suspicion of abnormality by the clinician. These couples were categorized as follows: couples with recurrent abortion and couples with recurrent abortion preceded by stillbirth or abnormal child (opted abortion). These cases were investigated by using G-banding karyotyping.

Result: In our study, heteromorphism of chromosome 9 was a ubiquitous finding in couples having recurrent abortions. In affected individuals/couples with chromosomal abnormalities in recurrent abortion cases, the female-to-male ratio was 5:2.

Conclusions: Chromosomal analysis is an important investigation for couples experiencing recurrent abortions. Heteromorphism of chromosome 9 is also an important finding in recurrent abortion. Genetic investigation, including karyotyping, would help in genetic counseling as well as decision-making and exploring further reproductive options.

## Introduction

Recurrent pregnancy loss (RPL)/abortion is a significant reproductive health issue, affecting approximately 1-2% of couples trying to conceive. It is defined as two or more consecutive pregnancy losses before 20 weeks of gestation [1]. Among the various etiological factors contributing to RPL, chromosomal abnormalities play a crucial role, accounting for 2-8% of cases [2,3]. These abnormalities can be classified into numerical and structural variations, including aneuploidies, translocations, inversions, and deletions, which may disrupt normal embryonic development and lead to pregnancy failure.

Parental chromosomal rearrangements, particularly balanced translocations and inversions, increase the risk of RPL by causing unbalanced gametes during meiosis. Additionally, embryonic chromosomal abnormalities, often arising de novo, are a major cause of early abortion. Advanced maternal age is a well-established risk factor for chromosomal errors, primarily due to meiotic nondisjunction.

Despite advancements in genetic testing, the precise mechanisms linking chromosomal abnormalities to RPL remain incompletely understood. Cytogenetic analysis, fluorescence in situ hybridization (FISH), and next-generation sequencing (NGS) have improved the detection of chromosomal aberrations, enabling better diagnosis and genetic counseling for affected couples. This study aims to investigate the prevalence and types of chromosomal abnormalities in couples with RPL, providing insights into their role in pregnancy loss and potential implications for clinical management.

## Materials and methods

Couples with recurrent abortion were referred to the cytogenetic laboratory from the Obstetrics and Gynecology OPD, AIIMS Raipur, for karyotyping. We have included only those who came between August 2023 and July 2025. These cases were non-consanguineous marriages. Consent was taken from patients visiting the cytogenetic laboratory. Each enrolled patient was identified by a unique patient identity number. Their demographic profile, medical history, associated risk factors or conditions that affect the pregnancy outcome, and menstrual, obstetric, and gynecological history were noted.

Inclusion criteria for the study were couples with recurrent abortion, couples with recurrent abortion preceded by stillbirth/abnormal child/healthy child, and couples aged between 18 and 45 years. Couples aged 46 years and above were excluded from the study.

Patients experiencing one abortion were also referred to the cytogenetic laboratory by clinicians for chromosomal analysis based on the clinical profile and previous records of the patients.

Both male and female spouses were karyotyped. For chromosomal analysis, we had taken 0.5 ml of heparinized peripheral blood, mixed with 5 ml of ready-to-use culture media Karyomix (HiKaryoXL RPMI medium with L-glutamine, fetal bovine serum (FBS), phytohemagglutinin M (PHA-M), penicillin, streptomycin, and sodium bicarbonate; HiMedia Laboratories, Thane, India). Cultures were incubated for 70 hours in 5% CO2 incubator at 37°C. Harvesting was done after 70 hours. Colchicine was used as a mitotic arrest agent. Giemsa trypsin banding (GTG) was done for metaphase chromosomes. In each case, 20-25 metaphases were microscopically examined and scored, and a karyotype was obtained in Metafer and Ikaros (Carl Zeiss, Oberkochen, Germany). In suspected cases, 50 cells were analyzed on an automated system (300-850 band levels of resolution). As our sample size was small, data analysis was done with percentage calculation.

## Results

Thirty couples were included in the study. Their age ranged from 26 to 39 years, with a mean age of 31.06 ± 2.54 years. These couples were categorized as follows: couples with recurrent abortion and couples with recurrent abortion preceded by stillbirth or abnormal child (opted abortion).

In this study, the highest number of patients were seen in the group with one to three miscarriages (21, 70%) (Table 1). The average age of females was 30.46 ± 3.04 years, and the average age of males was 33.23 ± 2.608 years. The average marriage age was 5.8 years. The number of previous miscarriages varied from one to six. Of the 84 pregnancies in 30 couples, 80 ended in spontaneous abortion, four ended in live birth, stillbirth, and Down syndrome. Some of them had a history of a previous fetus with Down syndrome.

**Table 1:** Grouping according to the number of abortions.

Number of abortions	Number of couples	Percentage (%)
1-3	21	70
4-5	7	23
6 or >6	2	7

**Figure 1:**
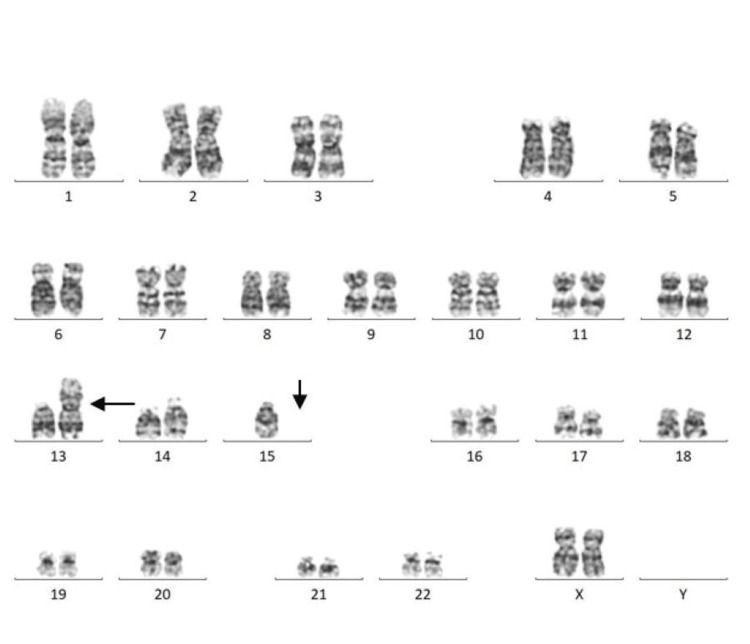
Female karyotype showing translocation between chromosomes 13 and 15 (46,XX,t(13;15)). Translocation is indicated by arrows.

**Figure 2:**
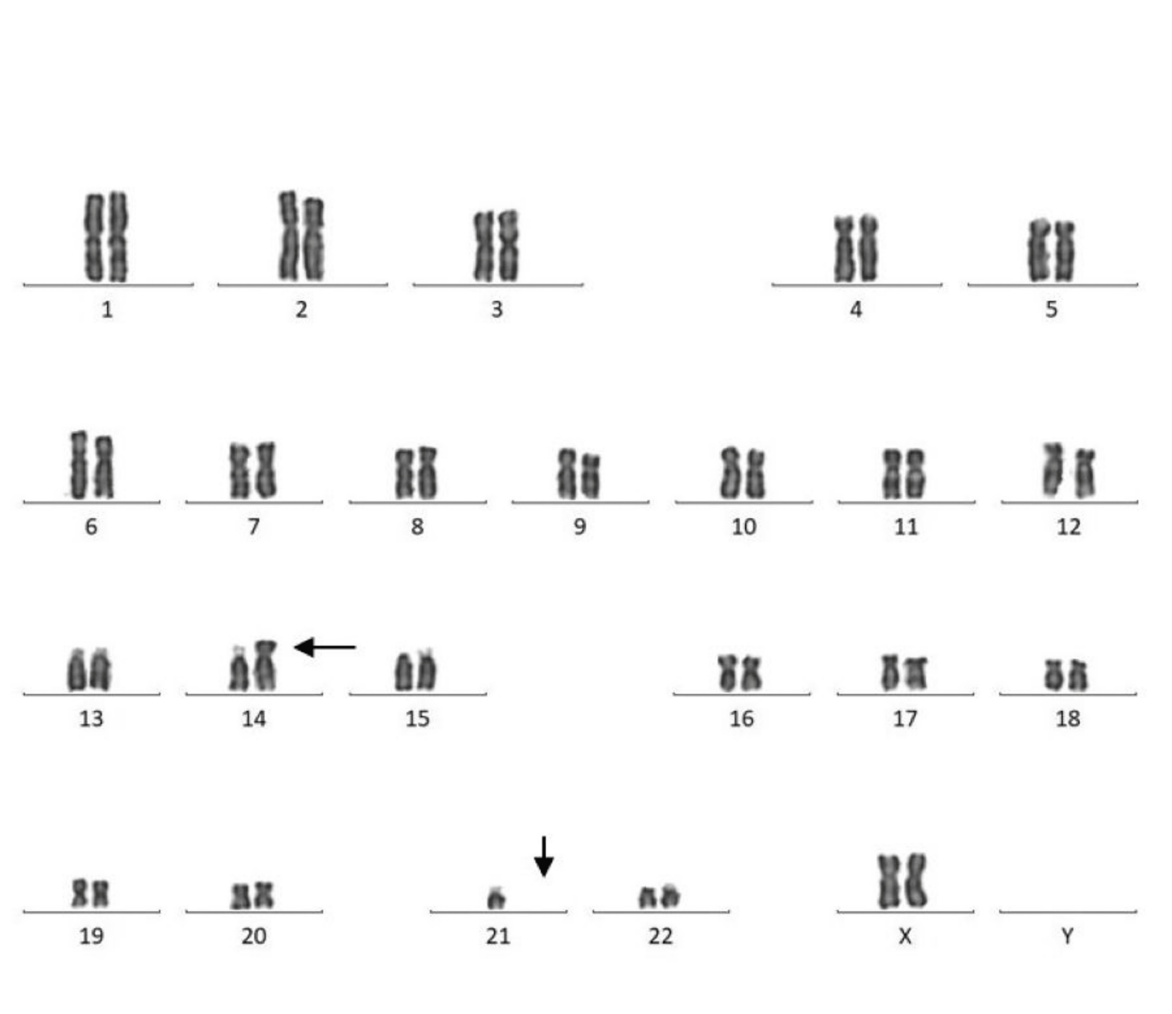
Female karyotype showing translocation between chromosomes 14 and 21 (46,XX,t(14;21)). Translocation is indicated by arrows.

**Figure 3:**
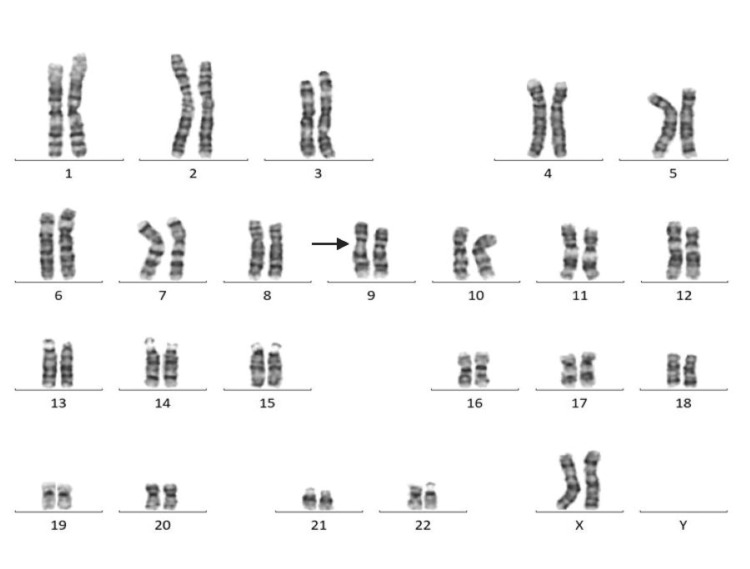
Female karyotype showing heteromorphism of chromosome 9 (46,XX,9qh+). Heteromorphism is indicated by an arrow.

**Figure 4:**
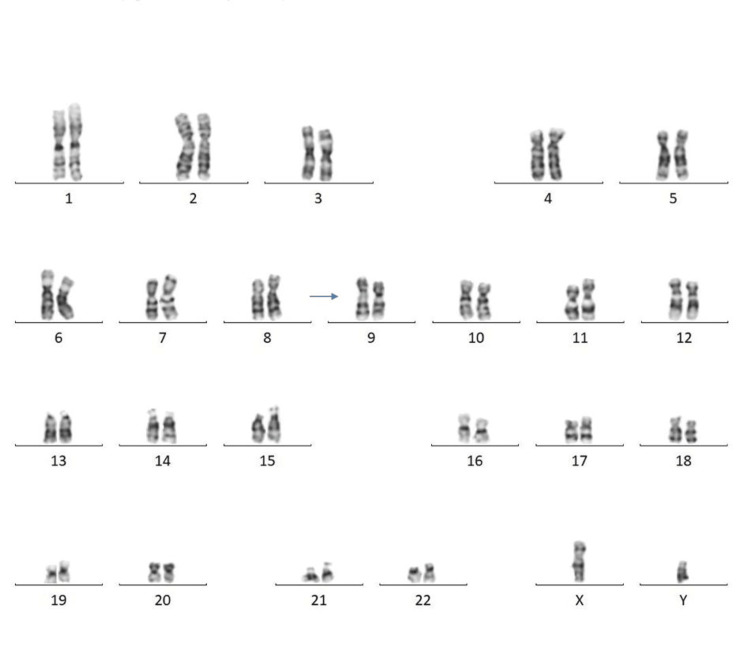
Male karyotype showing heteromorphism of chromosome 9 (46,XY,9qh+). Heteromorphism is indicated by an arrow.

**Figure 5:**
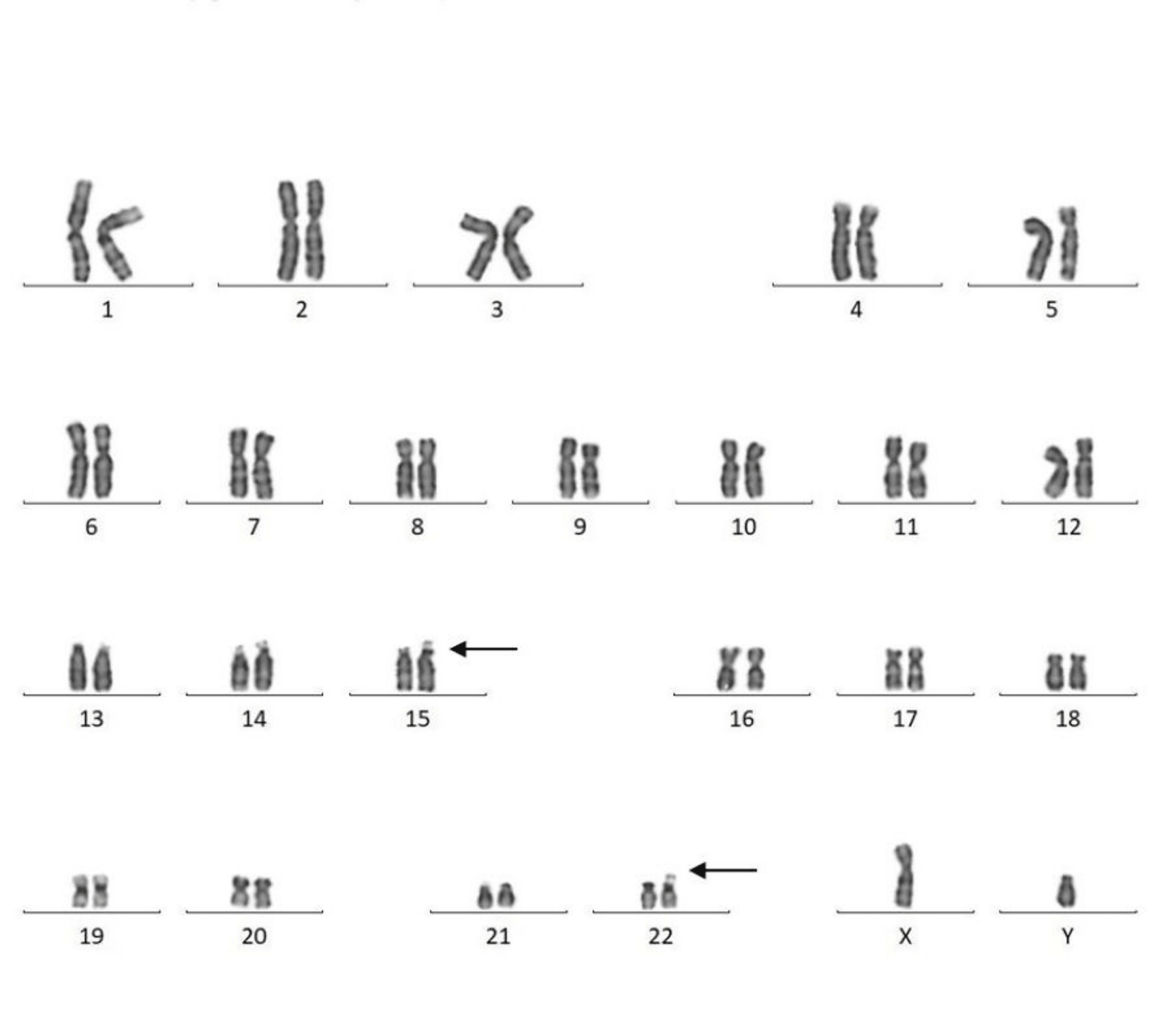
Male karyotype showing heteromorphism of chromosomes 15 and 22 (46,XY,15s+,22s+). Heteromorphisms are indicated by arrows.

Among 30 couples/60 individuals, chromosomal abnormalities were detected in seven individuals (11.6%). In the current study, five (71.42%) females and two (28.57%) males were found to have abnormal karyotypes. Chromosomal abnormalities were found in two (28.57%), and chromosomal variants were found in five (71.42%) cases. Among seven cases, structural abnormalities (Robertsonian translocation) were observed in two cases (Figures 1, 2) involving chromosomes 13 and 15 (D/D group) and 14 and 21 (D/G group) (Table 2). Heteromorphism of chromosome 9 was observed in four cases: three in females and one in a male (Figures 3, 4). Heteromorphism of chromosomes 15 and 22 was observed in one case (Figure 5).

**Table 2:** Cases with structural abnormalities and number of abortions.

Cytogenetic finding	Number of abortions	Sex	Age (year)
46,XX(9p11-q13)	3	Female	31
45,XX,der(13;15)(q10;q10)	3	Female	38
46,XX,9qh+	1	Female	27
45,XX,t(14;21)	2	Female	28
46,XX,9qh+	1	Female	29
46,XY,9qh+	1	Male	30
46,XY,15s+,22s+	2	Male	31

## Discussion

Among fertile women, recurrent abortion, which is a difficult medical problem, occurs in approximately 1-2% [4]. Cytogenetics is an important parameter for the medical evaluation of RPL.

The incidence of recurrent abortion in India was reported to be 10% [5]. The prevalence of parental chromosomal abnormalities observed in the current study was observed in six couples (20%) (seven individuals, 11.6%). This was greater than that reported by Saxena et al. (2012) [6] (2.78%), Goud et al. (2009) [7] (6.8%), Pal et al. (2009) [8] (8.9%), Pal et al. (2018) [5] (9.8%), and Niroumanesh et al. (2011) [9] (12%).

The incidence of recurrent abortion among patients in different age groups was as follows: 18-22 years (0%), 23-27 years (26.6%), 28-32 years (46.6%), and >33 years (26.6%). Whereas Patki et al. [10] found the incidence of RPL in age groups of 18-22 years, 23-27 years, 28-32 years, and >33 years to be 9.38%, 4.35%, 6.64%, and 14.68%, respectively. In our study, the age group of 28-32 years had the maximum incidence, whereas in Patki et al.'s study, the maximum incidence was in those older than 33 years [10].

Chromosome 9 heteromorphism emerged as the most common abnormality (57.14%), followed by Robertsonian translocations (28.57%) and heteromorphism on chromosomes 15 and 22 (14.28%). In our study, among the total seven cases, four cases (57.14%) had chromosome 9 heteromorphism, two cases (28.57% ) had Robertsonian translocation, and one case (14.28%) had chromosomes 15 and 22 heteromorphism. In our study, the female-to-male ratio was 5:2; however, it was not statistically significant. In comparison, Niroumanesh et al. [9] reported a ratio of 1.4:1, Sheth et al. [11] observed a ratio of 2.1:1, while Sudhir et al. [12] documented a ratio of 1:1.5.

Heteromorphism of chromosome 9 was the most common finding (4, 57.14%) in our study. This is a normal polymorphic variant but has an association with RPL. Studies suggested the mechanism of meiotic disruption in the heteromorphic variant of chromosome 9; the region on chromosome 9p11-q13 can interfere with chromosome pairing during meiosis, leading to inappropriate segregation and formation of improper gametes. Affected gametes during the formation of embryos have chromosomal imbalances contributing to pregnancy loss/abortion. Another common chromosomal abnormality of chromosome 9 is pericentric inversion. This may disrupt the gene function or regulatory regions critical for early embryonic development. Though 6-8% of healthy individuals have heteromorphism of chromosome 9, this is significantly associated with recurrent abortion [13].

In translocations, a Robertsonian translocation is the translocation of the long arm between two acrocentric chromosomes. In the current study, we found two cases (28.57%) having Robertsonian translocation. We had not found a translocation other than acrocentric chromosomes. In studies by Goud et al. [7], Pal et al. [5], Pal et al. [8], and Niroumanesh et al. [9], reciprocal translocation was the most common finding at 86%, 60%, 47%, and 31%, respectively. Other findings in recurrent abortions were marker chromosome, pericentric inversion, mosaicism, heteromorphisms, and satellite association in many studies (Table 3).

**Table 3:** Chromosomal abnormalities in recurrent pregnancy loss in different studies. sSMCs: small supernumerary marker chromosomes; Del: deletion; Dup: duplication; Aneu: aneuploidy.

Author	Place of study	Abnormal	No. of couples/cases	Chromosomal rearrangements					Total
				Reciprocal translocation	Robertsonian translocation	Pericentric inversion	Heteromorphism of chromosome 9	Others	
Current study	Chhattisgarh, India	7	30/60	-	02 (28.57%)	-	4 (57.14%)	1 (14.28%), heteromorphism of 15 & 22	7
Goud et al. (2009) [7]	Oman	26	380/760	18 (85.7%)	3 (14.3%)			Aneu: 1, Mosaic: 4	26
Pal et al. (2009) [8]	Malaysia, Bharu, Kelantan	5	56	3 (60%)	1 (20%)	-	-	1 (20%) mosaic Down	5
Dutta et al. (2011) [2]	Southern region of India	78	1162/2324	21 (26.92%)	6 (7.69%)	2 (2.56%)	44 (56.41%)	Del: 2; Dup: 1; Mark: 1; Aneu: 1	
Niroumanesh et al. (2011) [9]	Tehran, Iran	13	100/200	04 (30.8%)	3 (23%)	3 (23%), 1 (7.7%) paracentric inversion	1 (7.7%)	1 (7.7%) marker	13
Sheth et al. (2013) [11]	Gujarat	170	2428/4859	42 (24.70%)	30 (17.6)	3 (8%)	78 (45.9)	Mosaic: 7 (4.1%); sSMCs: 8 (4.7%)	Microdeletion: 1 (0.6)
Sudhir et al.(2016) [12]	Amritsar, Punjab	131	440/880	8 (6.10%)	1 (0.76%)	1 (0.76%)	Yqh+ 3 (2.29%); 15ps+ 116 (88.54%)	2 Dup (1.52%)	
Pal et al. (2018) [5]	Maharashtra, India	17	172/344	08 (47.05%)	02 (11.76%)	05 (29.41%)	-	2 (11.76%)	17

Studies observed that the most common abnormalities include balanced translocation and inversion, which have no consequence in the carrier, but in pregnancy, there is a risk of a fetus with unbalanced chromosomal abnormalities, resulting in RPL or even the birth of an abnormal baby. To optimize the counseling of RPL couples, it is advised to recommend or offer prenatal diagnostic screening to avoid the birth of a baby with chromosomal disorders [14].

In the current study, all couples were of non-consanguineous marriages. Researchers found a significant correlation and association of RPL with consanguineous marriage [15,16].

Genetic testing plays a very important role in RPL. These include karyotyping at the very first position. Karyotyping of both the male and female members of the couple may modify the treatment plan. Identification of chromosomal abnormalities helps them with appropriate counseling and management.

Limitations

The sample size of the study was small, and the study was conducted in a tertiary care center.

## Conclusions

The findings of the present study highlight the significant role of cytogenetic abnormalities in recurrent abortions. The prevalence of parental chromosomal abnormalities was higher (20% couples; 11.6% individuals) than most previously reported Indian studies, indicating a possible regional or cohort-related variation. The majority of affected individuals were within the 28-32 years age group, supporting advanced reproductive age as a contributory factor to recurrent abortion. Though heteromorphisms such as chromosome 9 variants are often considered normal polymorphic variants, their significant association with recurrent abortions in this study suggests a pathogenic contribution through meiotic disruption and chromosomal imbalance in gametes. The detection of Robertsonian translocations further reiterates the established risk of unbalanced gametes leading to fetal loss.

The female-to-male ratio of cytogenetic abnormalities (5:2) aligned with trends reported in earlier studies. Notably, all couples in this cohort were from non-consanguineous marriages, in contrast to other studies that found a positive association between consanguinity and recurrent abortion. Overall, this study emphasized the importance of cytogenetic evaluation, particularly karyotyping of both partners, at the earliest stage of recurrent abortion workup. Identifying chromosomal abnormalities not only aids in understanding the etiology but also facilitates targeted genetic counseling, risk assessment for future pregnancies, and appropriate prenatal diagnostic interventions to improve reproductive outcomes.
